# Simultaneous Identification of Multiple Causal Mutations in Rice

**DOI:** 10.3389/fpls.2016.02055

**Published:** 2017-01-17

**Authors:** Wei Yan, Zhufeng Chen, Jiawei Lu, Chunjue Xu, Gang Xie, Yiqi Li, Xing Wang Deng, Hang He, Xiaoyan Tang

**Affiliations:** ^1^College of Life Sciences, Capital Normal UniversityBeijing, China; ^2^Shenzhen Institute of Molecular Crop DesignShenzhen, China; ^3^College of Life Sciences, Peking UniversityBeijing, China; ^4^Guangdong Provincial Key Laboratory of Biotechnology for Plant Development, College of Life Sciences, South China Normal UniversityGuangzhou, China

**Keywords:** SIMM, next-generation sequencing technology, single nucleotide polymorphism, SNP index, allele index, Euclidean distance, sequence correction

## Abstract

Next-generation sequencing technologies (NGST) are being used to discover causal mutations in ethyl methanesulfonate (EMS)-mutagenized plant populations. However, the published protocols often deliver too many candidate sites and sometimes fail to find the mutant gene of interest. Accurate identification of the causal mutation from massive background polymorphisms and sequencing deficiencies remains challenging. Here we describe a NGST-based method, named SIMM, that can simultaneously identify the causal mutations in multiple independent mutants. Multiple rice mutants derived from the same parental line were back-crossed, and for each mutant, the derived F2 individuals of the recessive mutant phenotype were pooled and sequenced. The resulting sequences were aligned to the Nipponbare reference genome, and single nucleotide polymorphisms (SNPs) were subsequently compared among the mutants. Allele index (AI) and Euclidean distance (ED) were incorporated into the analysis to reduce noises caused by background polymorphisms and re-sequencing errors. Corrections of sequence bias against GC- and AT-rich sequences in the candidate region were conducted when necessary. Using this method, we successfully identified seven new mutant alleles from Huanghuazhan (HHZ), an elite *indica* rice cultivar in China. All mutant alleles were validated by phenotype association assay. A pipeline based on Perl scripts for SIMM is publicly available at https://sourceforge.net/projects/simm/.

## Introduction

Forward genetics based on EMS mutagenesis and cloning of mutant genes are of fundamental importance to the understanding of plant gene functions. Traditional map-based cloning approach has been widely used for cloning of mutant plant genes. It involves construction of mapping population(s) by crossing the mutant plant with wild-type plant of extensive genomic polymorphisms (Lukowitz et al., 2000). DNA sequence polymorphisms, such as simple sequence repeats, SNPs, and short insertions or deletions, are often used as markers for determination of parental chromosomal locations in the mapping population (Peters et al., 2003). By identifying markers that are tightly associated with the mutant phenotype, chromosomal region harboring the causal mutation can be identified. The mutant gene is further defined by sequencing the chromosomal region and functional complementation (Lukowitz et al., 2000; Jander et al., 2002). Despite the wide application, map-based cloning is labor-intensive and time-consuming. In addition, it is not suitable for cloning of mutant genes with ambiguous phenotypes in the mapping population.

The rapid development of NGST renders the sequencing of plant genomes routine. Consequently, several NGST-based methods have been developed for cloning of the mutant genes, including SHOREmap (Schneeberger et al., 2009), NGM (Austin et al., 2011), MutMap (Abe et al., 2012), MutMap+ (Fekih et al., 2013), and NIKS (Nordstrom et al., 2013).

Like map-based cloning, SHOREmap and NGM both involve crossing of the mutant with a remotely related wild-type plant; the derived F2 plants of the mutant phenotype are bulk-sequenced. Both methods have to deal with the numerous background polymorphisms between the two crossing accessions, which render it difficult to determine causal mutations. Thus far, SHOREmap, NGM, and other related methods have been successful only for cloning of mutant genes in Arabidopsis (Schneeberger et al., 2009; Austin et al., 2011; Uchida et al., 2011).

MutMap (Abe et al., 2012) takes a different approach by back-crossing the mutant with the wild-type parent, and 20–30 derived F2 individuals of the mutant phenotype are bulk-sequenced. MutMap requires sequencing and construction of a pseudo-genome for the wild-type parent prior to sequence alignment with the bulk sequence of the mutant plant. The pseudo-genome is generated by aligning the genome sequences of the wild-type parent with an assembled reference genome and then replacing the SNPs. Because MutMap deals with only the SNPs incorporated by mutagenesis, many fewer sequence polymorphisms are encountered in sequence alignment. Methods related to MutMap have been successfully used for cloning of mutant genes in Arabidopsis and rice (Abe et al., 2012; Zhu et al., 2012; Allen et al., 2013; Takagi et al., 2013).

Unlike MutMap, MutMap+ (Fekih et al., 2013) does not require backcross of the mutant with the wild-type parent. Instead, a single M2 heterozygous plant is selfed, and the derived M3 plants of the mutant and wild-type phenotypes are bulk-sequenced and then respectively aligned to the pseudo-genome of the wild-type parent. MutMap+ is particularly useful for cloning of mutant genes causing lethal or infertile phenotype that are not amenable for crossing.

NIKS (Nordstrom et al., 2013) is a reference-free algorithm that can be used for discovery of causal mutations even in plants that do not have an assembled reference genome sequence. It relies on the analysis of *k-*mers, which are defined as sub-sequences of length *k* of a sequencing read. The plants to be bulk-sequenced are generated as described for MutMap or MutMap^+^. NIKS starts by assessing the frequency of each *k*-mer within the bulk sequencing data. *k*-mers of identical sequences are saved as seeds for further comparison with the sequence of the wild-type parent, while *k*-mers of polymorphic sequences are discarded. In theory, NIKS can be applied to identify mutant genes that are caused not only by SNPs but also by small indels and large deletions.

The aforementioned NGST-based methods significantly accelerate the cloning of mutant genes in plants. However, since these methods depend on the comparison of the mutant bulks with wild-type bulks, they frequently produce dozens of candidate sites and sometimes fail to identify causal mutations (Nordstrom et al., 2013). There is a clear need for a method that can better resolve causal mutations. Here we report a method named SIMM for precise identification of multiple causal mutations in rice. SIMM does not need the genome sequence of the wild-type parent as reference. It can resolve candidate mutations to one or a few SNPs. Applications on seven published rice mutants and seven HHZ mutants indicated that SIMM is a proficient method which can simultaneously resolve the causal genes in multiple mutants.

## Materials and Methods

### EMS Mutagenesis and Mutant Screening

Sixty kilograms of HHZ seeds were soaked in water for 22 h and then drained. The drained seeds were incubated with 0.7% EMS solution at 28°C for 12 h and stirred every 30 min. The seeds were then washed five times with water, each time for 10 min, and then washed with running water for 1.5 h. The seed germination rate was determined to be ∼75%. Mutagenized seeds were cultivated in field, and seeds from ∼1,000 plants were pooled. Approximately 40,000 grains from each pool were planted, and mutants of various abnormal phenotypes were screened from 50 pools of the mutant library.

### Genetic Analysis of Mutants and Construction of Sequencing Population

Fertile mutants of abnormal phenotype were allowed to set seeds. For each mutant, 40 seeds were planted for phenotype confirmation. One plant with an inheritable phenotype was chosen to back-cross with wild-type HHZ, and the derived F1 plants were further selfed to produce F2 seeds. Sterile mutants were directly crossed with wild-type HHZ to produce F1, which then selfed to produce F2 seeds. Phenotype segregation ratios were evaluated with 160 F2 plants, and those displaying 3:1 segregation of wild-type to mutant plants were regarded as recessive mutants. Thirty F2 individuals of the mutant phenotype were selected for DNA extraction.

### DNA Extraction and Sequencing

Total DNA was extracted using DNeasy Plant Mini Kit (Qiagen) according to the manufacturer’s protocol and quantified using a Nanodrop^TM^ 2000 spectrophotometer (Thermo Scientific). Equal amounts of DNA from each plant were pooled, and 5 μg of the pooled DNA was used for preparation of a sequencing library of average insert size 200–500bp, according to the protocol for the Paired-End DNA Sample Prep Kit (Illumina). The library was sequenced to 25∼40x of genome coverage with the Illumina Hiseq 2000 platform.

### Correction of Sites of Low Coverage Depth or Low Quality

A mutant with defined candidate regions but no identifiable causal mutations was subjected to site correction of quality and coverage depth based on the GC content. Correction was applied only to those sites with base quality <20 or depth between 5 and 15. Site correction was performed according to following rules: 1, The average depth 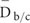 for each GC content group is calculated as 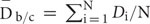, where *N* is the number of non-overlapping 200 bp bins randomly selected from the background regions (*b*) and candidate regions (*c*). The maximal base depth is limited to 300 to minify the influence of repetitive regions on calculation of the average depth. 2, The average quality 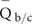 for each GC content group is calculated as 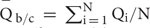. 3, The depth coefficient (*d*) and quality coefficient (*q*) are respectively calculated as 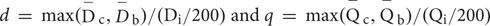. 4, The quality coefficient or depth coefficient was multiplied with the actual base quality or actual base depth in the same GC content group to get the corrected quality or corrected depth for each site. The corrected values were then used for SNP calling and calculation of SNP index and ED values.

### High Resolution Melting Analysis

Total DNA was extracted from rice leaves using a modified CTAB method (Allen et al., 2006) and diluted to 100 ng/μl. PCR reactions contained final concentrations of 0.05 U/μl Taq DNA polymerase (Takara), 1x PCR buffer, 25 μM dNTP each, 0.1 μM each primer, 5 ng/μl DNA template, 0.2x EvaGreen (Biotium), and were made up to 10 μl with water. Reactions were cycled on a thermal cycler (94°C 3 min, 40 cycles of [94°C 30 s, 58°C 30 s, 72°C 10 s], 72°C 5 min, 95°C 1 min, 25°C 1 min) and internal temperature calibrators were added into the amplification products. HRM data were collected on a LightScanner (Idaho Technology) (53∼98°C, hold at 50°C) and analyzed with the LightScanner Call-IT software (Idaho Technology).

## Results

### Principle of SIMM

**FIGURE 1:**
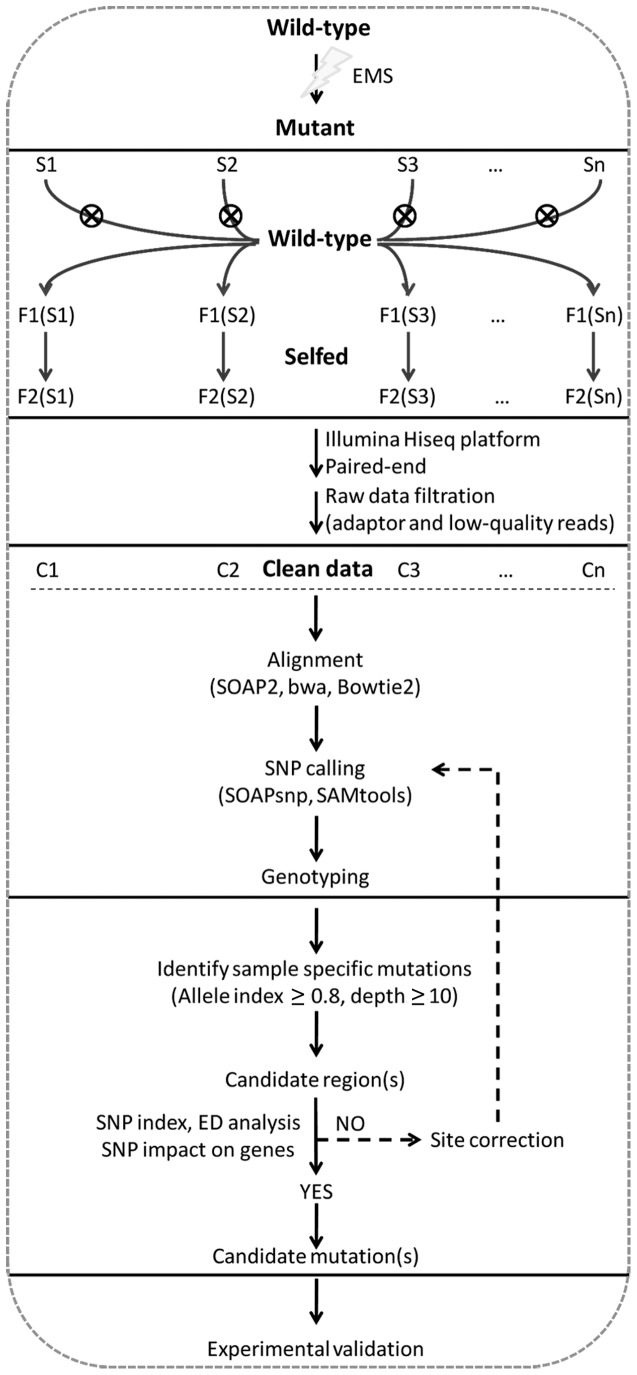
**Simultaneous identification of multiple mutations analysis pipeline.** Multiple rice mutants generated from the same parental line by EMS mutagenesis are back-crossed to generate F1, and then selfed to produce F2. Equal amounts of DNA from 20 to 30 F2 mutant individuals are pooled and used for construction of a paired-end library with insert size of 200∼500 bp. Libraries are sequenced to 20∼40x of genome coverage using the Illumina Hiseq platform. Adaptor sequences and low-quality reads are removed, and clean reads are aligned to the reference genome with SOAP2, bwa, or Bowtie2. SNPs are called using SOAPsnp (for SOAP2) or SAMtools (for bwa and Bowtie2). SNPs specific for the test mutant are identified by an AI ≥ 0.8. Candidate mutations are identified by considering SNP index, ED^6^, and potential impact on gene function. The candidate mutations are then experimentally validated with HRM. If no causal mutation is identified in the candidate region, site correction is conducted on sites with low quality or depth based on GC content. The corrected sites are then re-analyzed to identify candidate mutations.

Simultaneous identification of multiple mutations was primarily designed to identify casual mutations generated by EMS mutagenesis by simultaneous analysis of multiple mutants derived from the same parental plant, without requiring a wild-type parental genome sequence as reference (**Figure 1**). Each discrete mutant was back-crossed with the wild-type parent, and 20–30 F2 individuals of the recessive mutant phenotype were pooled and sequenced to >20x of the genome size using the Illumina Hiseq 2000 platform.

The raw short reads were first cleaned of adapter sequences, low quality reads (average Phred quality < 20), and reads containing > 10% Ns using Cutadapt (Martin, 2011). Clean reads for each sample were then aligned to the Nipponbare reference genome (Release 7 of the MSU Rice Genome Annotation Project)^1^ using software SOAP2 (Li et al., 2009b), bwa (Li and Durbin, 2009), or Bowtie2 (Langmead and Salzberg, 2012). Reads that aligned to two or more chromosomal positions were discarded due to the uncertainty of their genomic origin. Only uniquely mapped reads were retained for SNP calling using SOAPsnp (Li et al., 2009a) (for SOAP2) or SAMtools (Li H. et al., 2009) (for bwa and Bowtie2). Total SNPs between each mutant bulk and Nipponbare were identified and further compared among the mutants to identify SNPs specific for each discrete mutant strain. SNPs with <5 supporting reads in the mutant were discarded, as those may have resulted from sequencing errors or insufficient clusters.

MutMap and MutMap+ both compare EMS mutants with the wild-type parental line (Abe et al., 2012; Fekih et al., 2013). However, sequencing deficiencies such as sequencing errors and insufficient clusters often introduce errors into this pair-wise comparison. To overcome this problem, we introduced an AI to assess SNPs supporting wild-type alleles in background mutants (i.e., all mutant strains except the test mutant). AI was calculated as the number of reads supporting the wild-type genotype divided by the total number of reads covering the site in the background mutants. A SNP was regarded as test strain-specific when AI of this site was ≥0.8, allowing 20% sequencing errors in background mutants. Test strain-specific SNPs with read depth ≥10 were further assessed with SI, which was calculated as the number of mutant reads divided by the number of total reads in the test mutant. SNP responsible for the mutant phenotype should have a SI = 1, but due to the potential of sequencing errors, we lowered the cutoff of SI to 0.8. Those sites with SI ≥ 0.8 were all retained as candidates.

Although SI was a good indicator of candidacy, there were often too many sites when using SI ≥ 0.8 as threshold. To further refine candidate SNPs, ED analysis (Hill et al., 2013) was employed, which considered the SNP index in the test strain and reference mutants. ED value was calculated as ED = $ED=\sqrt{2\times {\left(SI_{t}-SI_{bc}\right)}^{2}}$, where *SI_t_* stands for the SI of mutation allele in the test strain, whereas *SI_bc_* stands for the SI of the same mutation allele in the background mutants. Since ED value ranged from 0 to $\sqrt{2}$, it was raised to power 6 (ED^6^), which was sufficient to enlarge the differences between causal mutations and closely linked mutations, and to signify candidate regions. Local linear regression curve (Loess fit) (Jacoby, 2000) was appended to show the distribution of SI and ED^6^ values along the chromosomes. Candidate regions harboring the causal mutation are expected to show a cluster of SNPs with high SI and ED^6^ values. Finally, the impacts of SNPs on gene function were evaluated. Mutations at intron splicing sites or non-synonymous mutations were ranked highest, followed by mutations in the promoter region, intron, and intergenic region.

If the aforementioned protocol correctly identifies the candidate region but fails to identify the causal mutation, we applied a correction of sites in the candidate region based on GC content. SNPs filtered out by quality and/or depth criteria were corrected according to the quality coefficient and/or quality coefficient, and then subjected to the SIMM pipeline. Ultimately, the candidate mutations were experimentally validated using phenotype association assay.

### Application of SIMM on Seven Previously Analyzed Rice Mutants

To evaluate the performance of SIMM, we first analyzed the bulk-sequencing data of seven rice mutants released by Abe et al. (2012). These data were analyzed using both MutMap (Abe et al., 2012) and NIKS (Nordstrom et al., 2013). MutMap revealed 101∼238 candidate sites with SI ≥ 0.9. Among them, 4∼24 were of CG to TA transitions common to EMS mutagenesis. NIKS revealed 7∼21 candidate mutations of CG to TA transitions, but the number of other mutation types was not reported. SIMM revealed 6∼32 candidate sites for each mutant with SI ≥ 0.8, of which 4∼17 were CG to TA mutations (**Table 1**). When ED analysis was applied, candidate sites for each mutant were reduced to 3∼15 (**Table 1**).

**Table 1:** Comparison of results from MutMap, NIKS and SIMM analyses on seven published mutants.

Sample	MutMap^1^	NIKS^2^	SIMM^3^	ED^4^	Chr	Locus	Genotype/aa	Candidate gene
Hit0746-sd	121 (13)	16	8 (8)	3	Chr8	27,272,776	G to A (D to N)	LOC_Os08g43120^b,c^
Hit5243-sm	229 (21)	19	32 (14)	15	Chr8	11,552,593	C to T (R to C)	LOC_Os08g19310^a,b,c^
Hit5500-sd	131 (9)	12	10 (4)	8	Chr9	20,062,177	T to A (L to stop)	LOC_Os09g33980^a,b,c^
Hit1917-pl1	101 (24)	21	19 (18)	7	Chr10	22,484,681	C to T (L to F)	LOC_Os10g41780^a,b,c^
						22,705,386	C to T (A to V)	LOC_Os10g42196^b,c^
Hit1917-sd	139 (10)	7	12 (12)	9	Chr12	23,278,301	G to A (splicing site)	LOC_Os12g37870^c^
						23,285,822	G to A (S to N)	LOC_Os12g37890^b,c^
Hit5814-sd	121 (4)	10	6 (4)	3	Chr4	23,555,382	A to T (R to stop)	LOC_Os04g39560^a,b,c^
						24,669,920	C to T (L to F)	LOC_Os04g41580^a,b,c^
						25,537,772	C to T (R to C)	LOC_Os04g43140^a,b,c^
Hit0813-pl2	238 (10)	20	6 (6)	4	Chr1	42,235,284	G to A (intron)	LOC_Os01g72800 ^b,c^
						42,282,527	C to T (intergenic)	–
						42,843,286	C to T (intergenic)	–
						43,121,603	G to A (intron)	LOC_Os01g74460^c^

*^1^is the number of candidate SNPs extracted by MutMap. Numbers in brackets indicate the GC to AT mutations. ^2^is the number of GC to AT mutations extracted by NIKS as candidate sites. ^3^is the number of candidate SNPs extracted by SIMM. Numbers in brackets indicate the GC to AT mutations. ^4^is the number of SNPs in candidate region defined by ED analysis, including all mutation types. ^a^indicates the candidate gene defined by MutMap. ^b^indicates the candidate gene defined by NIKS. ^c^indicates the candidate gene defined SIMM.*

The candidate sites were further evaluated of functional impacts on the genes, resulting in a final list of 12 candidate genes for these mutants, six of which were missed when using MutMap, and two were missed when using NIKS (**Table 1**). Candidate genes revealed by SIMM and NIKS were mostly identical except for the mutants Hit1917-sd and Hit0813-pl2. The candidate site identified by MutMap in mutant Hit1917-sd was identified neither by NIKS nor by SIMM (Abe et al., 2012; Nordstrom et al., 2013). SIMM identified two candidate sites for Hit1917-sd; one of them was also identified by NIKS, but the other one at the intron splicing site of *LOC_Os12g37870* was missed by NIKS (**Table 1**). MutMap did not detect either of the two sites (**Table 1**). *LOC_Os12g37870* is orthologous to *AT1G09430* encoding an ATP-citrate lyase. Arabidopsis plants expressing *AT1G09430* antisense RNA exhibit dwarfism (Fatland et al., 2005), which was consistent with the dwarf phenotype of Hit1917-sd (Abe et al., 2012). Based on these observations, we propose *LOC_Os12g37870* as the potential mutant gene for Hit1917-sd. For the pale green mutant Hit0813-pl2, NIKS detected an SNP in the intron of *LOC_Os01g72800*, a chloroplast SRP receptor. SIMM discovered this SNP and three other mutation sites, one in the intron of *LOC_Os01g74460*, and two in intergenic regions. Significantly, the orthologous gene of *LOC_Os01g74460* in Arabidopsis (*AT2G28390*) encodes a chloroplast-located protein. We consider both *LOC_Os01g72800* and *LOC_Os01g74460* to be prime candidates for the pale green phenotype of mutant Hit0813-pl2. These results indicated that SIMM has higher sensitivity and specificity than the other two methods in localizing the candidate genes.

### Identification of HHZ Mutant Genes Using SIMM

**FIGURE 2:**
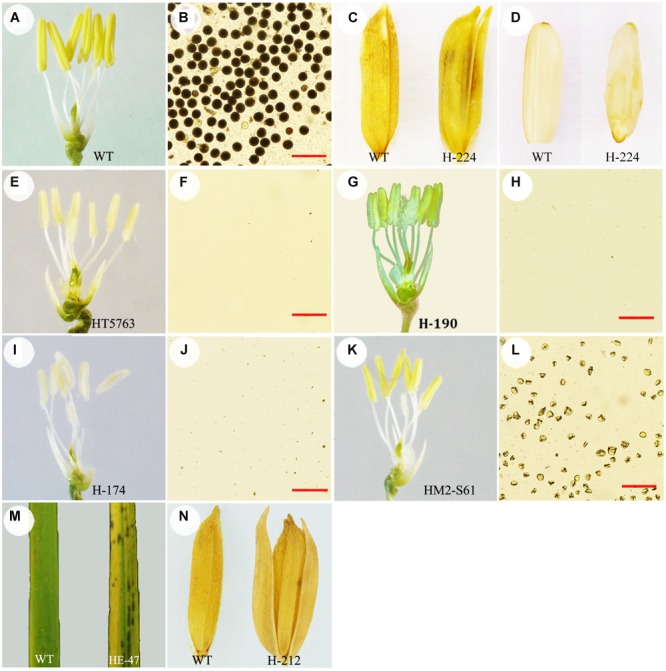
**Phenotype of seven HHZ mutants. (A,B)** wild-type HHZ; **(C,D)** H-224; **(E,F)** HT5763; **(G,H)** H-190; **(I,J)** H-174; **(K,L)** HM2-S61; **(M)** HE-47; **(N)** H-212. Palea and lemma were removed to show the anthers for the male sterile mutants. Pollen grains in **(B,F,H,J,L)** were released from the anthers and stained with 1% I_2_-KI solution. Seed, husked seed, and leaf from wild-type HHZ (WT) are shown in **(C,D,M,N)** as references. Bar = 100 μm.

We obtained seven HHZ mutants that are controlled by single recessive genes (**Figure 2**). We first used the MutMap method to identify the causal mutations, so the wild-type HHZ was sequenced to ∼10x of genome coverage. The bulked sequences of each mutant and wild-type HHZ were respectively aligned to the Nipponbare reference genome, which generated a total of 1.21–1.61 × 10^6^ SNPs for the mutants and 1.00 × 10^6^ SNPs for wild-type HHZ (**Table 2**, #SNPs^1^). We then compared the SNPs of each mutant against wild-type HHZ to identify the SNPs specific for each mutant, resulting in a total of 5,108∼9,230 specific SNPs (**Table 2**, #SNPs^2^), of which, 955∼1,546 had SI ≥ 0.8 (**Table 2**, #SNPs^2^, numbers in bracket). These SNPs were presumably the candidate mutations. However, it was not possible to pinpoint causal mutations from so many candidates.

**Table 2:** Summary of SIMM analysis of HHZ mutants.

Samples	Total reads	Aligned reads	Depth	Coverage (%)	#SNPs^1^	#SNPs^2^	#SNPs^3^	#SNP^4^	Candidate gene	Locus /genotype(aa)	SNP index	ED^6^
WT HHZ	121,453,146	107,052,411	10X	83.41	1,004,213	/	/	/	/	/	/	/
H-224	141,304,146	110,556,122	29X	88.53	1,564,462	6,063 (1,546)	1,109 (41)	9	LOC_Os03g43670 (*OsCR4*)	24,417,520 G to A (G to D)	0.9778	6.9913
HT5763	127,424,446	103,501,527	28X	88.18	1,308,965	9,230 (1,035)	6,486 (97)	4	LOC_Os04g39470 (*MYB80*)	23,511,644 G to A (E to K)	0.9231	4.9506
H-190	144,014,516	111,948,542	35X	87.36	1,210,418	5,108 (1,238)	330 (7)	7	LOC_Os03g58600 (*MEL1*)	33,373,677 T to G (Y to D)	1.0000	7.2970
H-174	154,449,224	118,039,787	37X	87.38	1,328455	5,505 (1,303)	573 (16)	13	LOC_Os03g58600 (*MEL1*)	33,372,744 G to A (E to K)	1.0000	7.9995
HM2-S61	173,294,984	131,445,408	41X	87.57	1,385,235	5,337 (1,399)	435 (10)	4	LOC_Os07g32480 (*Brk1*)	19,329,232 G to A (G to S)	0.9655	6.4798
HE-47	145,091,780	110,757,680	29X	89.16	1,609,185	5,217 (955)	1,051 (52)	8	LOC_Os03g06410 (*OsEDR1*)	3,206,988 T to C (S to F)	1.0000	7.9995
H-212	253,627,768	114,774,504	28X	90.17	1,286,823	7,012 (1,230)	3,109 (233)	20	LOC_Os07g04670 (*G1*)	2,068,093 C to T (Q to stop)	1.0000	7.9995

*^1^is the number of SNPs between the sequenced sample and Nipponbare reference genome (MSU v7). ^2^is the number of SNPs between the wild type HHZ and each of the mutants. Numbers in brackets are the SNPs with SNP index ≥ 0.8. ^3^is the number of SNPs specific for each mutant when referenced with all other mutants. Numbers in brackets are SNPs with SNP index ≥ 0.8. ^4^is the number of SNPs in the candidate region defined by ED analysis.*

We then took the SIMM approach by comparing the SNPs of one mutant strain against the SNPs of all other mutant stains, which generated 330∼1,109 SNPs specific for each mutant (**Table 2**, #SNPs^3^), except for the mutant H-212 which was contaminated by fungal DNA, and HT5763 that contains large unique genomic regions absent in other mutant strains. SNP index analysis identified 7∼233 candidate SNPs with SI ≥ 0.8 and read depth ≥ 10 (**Table 2**, #SNPs^3^, numbers in bracket), which were much smaller than those identified with MutMap (**Table 2**, #SNPs^2^, numbers in bracket). ED analysis further refined the candidate regions and narrowed down to 4–20 candidate sites for these mutants (**Table 2**, #SNPs^4^). Our initial functional assessment pinpointed one causal gene for each of these mutants, except H-212 (**Table 2**).

**FIGURE 3:**
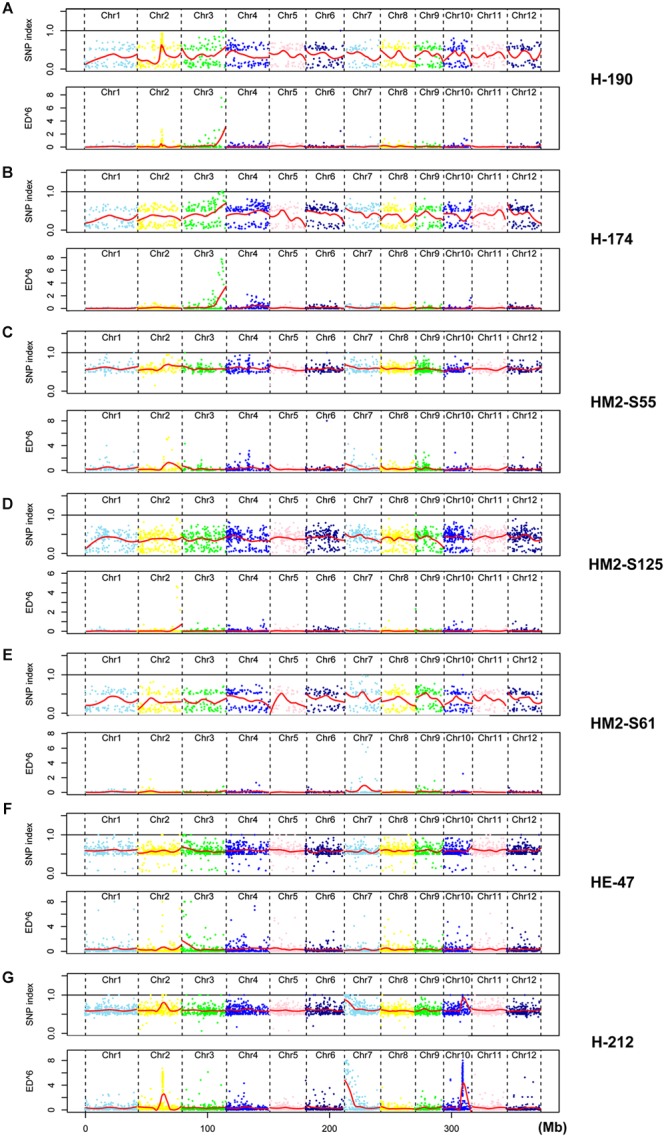
**Comparative plots of SNP indices and ED^6^ values for seven HHZ mutants. (A)** Mutant H-224. **(B)** Mutant HT5763. **(C)** Mutant H-190. **(D)** Mutant H-174. **(E)** Mutant HM2-S61. **(F)** Mutant HE-47. **(G)** Mutant H-212. Red lines in all plots represent Loess curve.

Mutant H-224 exhibited abnormal spikelet and seed morphology (**Figures 2C,D**). The palea and lemma were not tightly interlocked, resulting in seeds with open beaks and hulls. A large portion of the spikelets and seeds exhibited brownish palea and/or lemma with poorly filled grain inside. SNP index analysis indicated three putative candidate regions (**Figure 3A**). However, ED analysis highlighted a region on chromosome 3 containing 9 SNPs of high scores (**Figure 3A**). Only the SNP (Chr3: 24,417,520) located in *LOC_Os03g43670* (*OsCR4*) causes amino acid substitution from Gly (GGC) to Asp (GAC) (**Table 2**). We could not find *OsCR4* mutants in rice, but RNAi inhibition of *OsCR4* expression led to abnormal spikelets and seeds with phenotypes similar to what we observed in H-224 mutant (Pu et al., 2012). The less significant peak on chromosome 10 probably represents a different phenotype in the sequenced F2 mutants.

Mutant HT5763 was a male sterile mutant (**Figures 2E,F**). Both SNP index and ED analyses identified multiple candidate regions harboring SNPs of high scores (**Figure 3B**). Regions located on chromosome 2, 3, and 9 have high SNP densities, giving rise to a much larger number of specific SNPs in HT5763 than in other mutants (**Table 2**). These regions are likely heterologous chromosomal fragments that were still segregating in the EMS-treated seed population. The left end of chromosome 11 has two SNPs of high scores; one is a synonymous mutation, and the other one is in an intergenic region. Another candidate region on chromosome 4 carries a SNP (Chr4: 23,511,644) in *LOC_Os04g39470* (*OsMYB80*) causing an amino acid substitution from Glu (GAG) to Lys (AAG) (**Table 2**). OsMYB80 is highly homologous to the Arabidopsis AtMYB80 (also called AtMYB103) transcription factor (Higginson et al., 2003). AtMYB80 is required for pollen development in Arabidopsis (Phan et al., 2011). Mutation of this gene in Arabidopsis leads to male sterility. Complementation of the Arabidopsis *myb80* mutant with the rice *OsMYB80* gene restores male fertility (Phan et al., 2012), suggesting the orthologous relationship between OsMYB80 and AtMYB80. However, the function of *OsMYB80* in rice has not been confirmed by mutational analysis. Mutant HT5763 is male sterile with no pollen grains, indicating that *OsMYB80* may regulate pollen development in rice. Mutant HT5763 provides a tool for functional study of *OsMYB80* in rice.

H-190, H-174, and HM2-S61 were three other male sterile mutants (**Figures 2G–L**). Both SNP index analysis and ED analysis identified only one prominent candidate region for these mutants. The candidate regions for H-190 and H-174 are at the end of chromosome 3 that harbor 7 and 13 candidate SNPs, respectively (**Figures 3C,D**). H-190 has a SNP (Chr3: 33,373,677) in the 15th exon of *LOC_Os03g58600* (*MEL1*) that causes amino acid substitution from Tyr (TAC) to Asp (GAC) (**Table 2**). H-174 has a SNP (Chr3: 33,372,744) in the 20th exon of the same gene that causes amino acid substitution from Glu (GAG) to Lys (AAG) (**Table 2**). *MEL1* encodes an ARGONAUTE protein regulating male and female germ cell development, and mutations of this gene was reported to induce male sterility in rice (Nonomura et al., 2007). The candidate region for HM2-S61 is on chromosome 7 that harbors 4 SNPs (**Figure 3E**). One of these SNPs (Chr7: 19,329,232) located in *LOC_Os07g32480* (*Brk1*) causes amino acid substitution from Gly (GGT) to Ser (AGT) (**Table 2**). *Brk1* encodes a spindle checkpoint kinase acting during rice meiosis. A mutant allele of this gene was reported to be male sterile (Wang et al., 2012), which is consistent with our observation.

Mutant HE-47 has numerous red spots on leaves (**Figure 2M**). Three regions harboring multiple SNPs of high scores were identified for this mutant. The regions on chromosomes 2 and 10 were dismissed because SNPs in these regions were densely distributed (**Figure 3F**), which may represent heterologous genomic regions that are still segregating in the EMS-treated seeds. One region at the end of chromosome 3 contains 4 SNPs of high ED^6^ scores, one of which (Chr3: 3,206,988) located in *LOC_Os03g06410* (*OsEDR1*) causes amino acid substitution from Ser (TCA) to Pro (CCA) (**Table 2**). *OsEDR1* encodes a MAPK kinase kinase with a function in plant defense signaling. A T-DNA insertion allele of this gene shows spontaneous lesions on rice leaves (Shen et al., 2011), the same phenotype as we observed in mutant HE-47.

### Applying Sequence Correction to Identify the Causal Mutation in H-212

Mutant H-212 exhibited longer sterile lemmas compared with wild-type HHZ (**Figure 2N**). Notably, this mutant showed abnormal re-sequencing data with significantly lower aligned ratios to the reference genome, and the base contents of reads were significantly different from what in other sequenced mutants. We randomly chose 100 unmapped reads and blasted in NCBI, which showed that most of these reads originated from *Fusarium*. To make use of the sequence data, sequences highly similar to the *Fusarium* sequences were discarded. Then 10 bp at the 5′ end and 5 bp at the 3′ end of both paired-end reads were trimmed from the retained reads, followed by filtration with average Phred quality ≥ 20. The remaining reads were then searched for the causal mutations.

Three candidate regions were identified on chromosomes 2, 7, and 10, respectively (**Figure 3G**). Regions on chromosomes 2 and 10 were dismissed due to high SNP density. Twenty SNPs were identified in the candidate region on chromosome 7, two of which caused non-synonymous mutations. However, all these mutations showed segregation when a large number of F2 individuals were analyzed in phenotype association assays, indicating none being the causal mutation.

**FIGURE 4:**
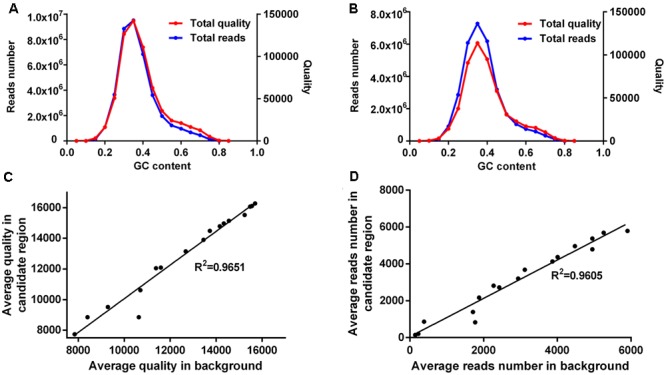
**Site corrections for mutant H-212. (A)** The total reads number (left *y*-axis) and total mapping quality (right *y*-axis) within the candidate region (Chr7: 2∼4 Mbp) are calculated for non-overlapping bins (200 bp) in each GC content group (increment 0.05). **(B)** Approximately 7,000 randomly selected bins from the whole genome are grouped based on their GC content. The total reads number (left *y*-axis) and total mapping quality (right *y*-axis) are calculated for bins in each GC content group (increment 0.05). The average quality **(C)** and average reads number **(D)** for bins in each GC content group are significantly correlated between the candidate region and background bins. Pearson’s correlation (*R*^2^) is calculated for each comparison.

Sequences generated by the Illumina Hiseq platforms tend to have low read coverage in GC-rich or AT-rich regions (Benjamini and Speed, 2012). We also found low mapping quality for these regions (**Figures 4A,B**). We speculated that the causal mutation was filtered out due to low read coverage and/or quality. Thus we re-evaluated the candidate region (Chr7: 2–4 Mb) for each mutant and corrected the base quality and depth for sites of base quality <20 or coverage depth between 5 and 15, based on GC content.

The candidate region was split into non-overlapping bins of 200 bp, and these bins were then grouped based on their GC contents (**Figure 4A**). For reference, ∼7,000 bins of 200 bp were randomly picked as background bins from other sequences of the same mutant and grouped according to their GC contents (**Figure 4B**). Total reads and total mapping quality were respectively calculated for each GC content group. The maximal base depth was set to 300 to minify the possible influences induced by repetitive regions. It is clear that, in both the candidate region and background bins, GC-rich and GC-poor bins have low read coverage as well as low mapping quality (**Figures 4A,B**).

The average mapping quality for bins in each GC content group was strongly correlated between the candidate region and background bins (**Figure 4C**), and so was the average read coverage (**Figure 4D**). Therefore, the bigger value of average quality or average read depth of the candidate region and background bins was chosen for calculation of quality coefficient or depth coefficient. Corrected quality or corrected depth was obtained by multiplying the quality coefficient or depth coefficient with the actual base quality or actual base depth for the site to be corrected, respectively. Sites with corrected quality or depth were then utilized for SNP calling and re-calculation of SNP index and ED^6^ values, resulting in 34 new SNPs in the candidate region. Five of them exhibited high SNP index and ED^6^ scores. One SNP located at Chr7: 2,068,093 induced a premature stop codon in *LOC_Os07g04670* (*G1*) (**Table 2**). *G1* encodes a transcriptional regulator. Yoshida et al. (2009) reported a *G1* mutant allele of long sterile lemma phenotype consistent with what we observed for mutant H-212.

### Phenotype Association Assay of the Candidate Genes

Phenotype association assay was conducted to validate the candidate genes in seven HHZ mutants via HRM analysis (Lochlainn et al., 2011) using primers in **Table 3**. As expected, all the individuals of mutant phenotype carried a homozygous mutation. Approximately two-thirds of the plants with a wild-type phenotype were heterozygous for the mutation, and one-third were homozygous for the wild-type genotype (**Table 3**). The mutations in our candidate genes are thus very tightly associated with the mutant phenotype. Because the phenotypes we observed in our mutants were consistent with what in the published studies, we propose that SIMM correctly identified the causal mutations for the seven HHZ mutants.

**Table 3:** Validation of causal mutations identified in HHZ mutants via co-segregation assay.

Sample	Phenotype	WT/Mutant	χ^2^ (3:1)	Candidate gene	Primers	WT		Mutant
						Homo	Hetero	Homo
H-224	Open hull and brownish palea/lemma	299:85	0.0188 (S)	LOC_Os03g43670	F: 5′-CAGCTGGTTCTGTGTTCAATTGTGGC-3′ R: 5′-CCTGCTCCTATAGACTGGAACCGCAC-3′	27	63	26
HT5763	Male sterile	232:80	0.0008 (S)	LOC_Os04g39470	F: 5′-CCACTCGTTGATGCCTACCTGTTGC-3′ R: 5′-GGCTGCGGTGGACCAACTACCTC-3′	39	72	30
H-190	Male sterile	512:264	0.1444 (NS)	LOC_Os03g58600	F: 5′-CATGTAGGTTGTGGCATC-3′ R: 5′-CCTGTCTATGTGGTTGAGC-3′	42	76	35
H-174	Male sterile	217:67	0.0043 (S)	LOC_Os03g58600	F: 5′-TGTGCTCTTGACATTCCT-3′ R: 5′-GCAAAGATTGTTGGTCAG-3′	21	58	25
HM2-S61	Male sterile	251:93	0.0083 (S)	LOC_Os07g32480	F: 5′-GCACCAAGGTGGTAGAAAG-3′ R: 5′-AGCAACCAGGTCTTCTGT-3′	24	55	32
HE-47	Red lesions on leaves	97:39	0.0257 (S)	LOC_Os03g06410	F: 5′-TGGAGTTGAATAGAGTGT-3′ R: 5′-GAATATCCCTCGTTTACT-3′	36	90	42
H-212	Long sterile lemma	132:44	0.0000 (S)	LOC_Os07g04670	F: 5′-TGCTCGCCGGCGGAGCTG-3′ R: 5′-GCGAGGTACTGCGTGAAG-3′	30	85	45

*S, significant; NS, not significant. WT/mutant indicates the number of F2 plants of wild-type and mutant phenotype. Primers are those used in HRM experiments to verify the candidate genes; Numbers in the “WT” and “mutant” columns indicate F2 plants of homozygous wild-type, heterozygous wild-type, and homozygous mutant genotypes shown by HRM experiment.*

## Discussion

The identification of causal mutations based on NGST provides an alternative approach for cloning of plant genes. Here we report a method that can simultaneously identify causal mutations in multiple mutants derived from the same parental plant. Different from the published methods such as MutMap (Abe et al., 2012) and NIKS (Nordstrom et al., 2013), SIMM does not need the sequence of the wild-type parent as reference, nor the assembly of sample specific *k*-mers. Instead, SIMM compares the test mutant with all other mutants as reference. This comparison scheme compensates for the sequencing deficiency in some mutant strains and avoids wrongly retaining or excluding candidate sites. As a result, SIMM can accurately resolve the causal mutation to many fewer candidate sites. The advantage of SIMM over MutMap and NIKS is shown by the analysis of seven published mutants in **Table 1**. For these mutants, the candidate sites resolved by SIMM were fewer but more complete than those resolved by MutMap or NIKS. Six candidate genes identified by SIMM were missed by MutMap, and two potentially valuable candidates were missed by NIKS as well (**Table 1**), In addition, MutMap yielded too many candidate sites for HHZ mutants, making it difficult to pinpoint the causal mutation. These results indicated that SIMM is more proficient than MutMap and NIKS in resolving candidate genes.

Two measures were included in the SIMM protocol to enhance accurate identification of causal mutations. First, all mutants were referenced for the identification of EMS-induced strain-specific SNPs. This is of key importance for SIMM to differentiate between causal mutations and background polymorphisms. Background polymorphisms between the test mutant and the wild-type parent are common, especially if the plants are derived from a recently established variety that still has many chromosomal segments heterogeneous in the population. Such background polymorphisms cannot be efficiently eliminated in pair-wise comparison between the mutant and wild-type genomes (as done in MutMap and NIKS), but can be eliminated or significantly reduced when multiple mutants are compared. SIMM cuts down the number of strain-specific SNPs to 6∼70% of those identified using MutMap (**Table 2**, #SNPs^2^ vs. #SNPs^3^). Thereby, a large number of background polymorphisms were excluded for subsequent analysis. Second, AI and ED analyses were introduced into the SIMM algorithm, which significantly reduced the background noises caused by sequencing deficiencies, and increased the reliability of genotyping the wild-type and mutant alleles for the test mutant. The application of AI and ED analyses reduced not only the number of candidate SNPs to be experimentally verified, but also the chance to wrongly exclude the causal mutations.

Sequences generated by the Illumina Hiseq platform usually tend to have low read coverage in GC-rich or AT-rich regions (Dohm et al., 2008; Benjamini and Speed, 2012). We also found low mapping quality for these regions. If the causal mutation happens to be in such a region, it would be filtered out by the cutoff criteria for coverage depth and sequence quality. This is exemplified by mutant H-212, in which a candidate region was correctly identified but the causal mutation was not found. Further examination of reads covering the candidate region revealed the presence of sequence bias. Corrections of read quality and coverage depth were performed to the sites filtered out due to the sequence bias, based on the GC content. Using the corrected quality and depth for SNP index and ED analyses, a potential causal mutation site was identified for mutant H-212.

**FIGURE 5:**
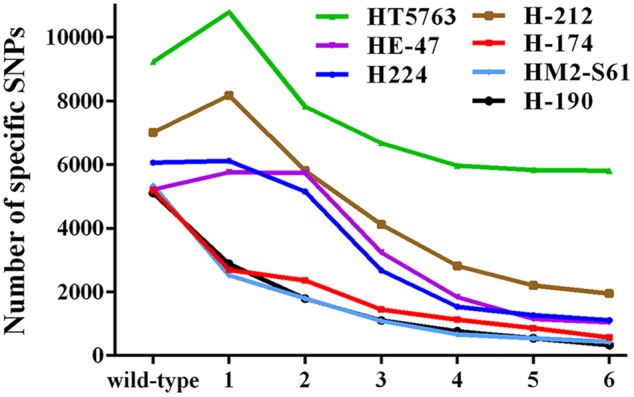
**Statistical analysis of the number of background mutants on the quantity of specific SNPs for each HHZ test mutant.** All mutants exhibit a larger number of specific SNPs when only using the wild-type HHZ as reference. The number of specific SNPs for each test mutant decreases dramatically when 3–5 mutants are used as reference. Numbers in the *x*-axis represent the number of reference mutants.

The SIMM method is particularly suitable when multiple mutants derived from the same parental plant are to be analyzed. We conducted a statistical analysis to assess the influence of the number of background mutants on the power of SIMM to resolve the strain-specific SNPs, based on the seven HHZ mutants. As shown in **Figure 5**, the strain-specific SNPs reduced dramatically when 3-5 background mutants were used. However, the resolution power did not increase significantly when five or more mutants were analyzed. Thus, sequencing 3–5 mutants is suggested for SIMM when dealing with mutants of less background polymorphisms. However, sequencing of more mutants is necessary if they have higher background polymorphisms. When only one or two mutants are available, then bulk sequencing of a few wild-type plants derived from the same parental line is recommended. The presence of a sibling mutant derived from the same mutant line can wipe out candidate regions for both siblings. However, such mutants can be recognized by the SIMM pipeline, which then analyzes them individually. Identical mutant alleles derived from independent mutagenesis events show overlapping candidate regions, and the SIMM pipeline can recognize such mutants and process them separately.

## Conclusion

Simultaneous identification of multiple mutations is a proficient method that can simultaneously resolve the causal genes in multiple mutants without the need to sequence the wild-type genome. By introducing AI analysis, ED analysis, and site correction, SIMM can accurately resolve the causal mutation to one or a few candidate sites. SIMM showed better sensitivity and specificity than MutMap and NIKS when analyzing seven published rice mutants. Applications on seven HHZ mutants further showed the efficacy of SIMM for NGST-based identification of mutant plant genes.

## Accession Numbers

Sequencing data can be found in the NCBI Sequence Read Archive under accession number SRP058039.

## Author Contributions

XD, HH, and XT conceived the ideas for the study; WY and HH designed the algorithm; ZC, JL, CX, GX, and YL performed the experiments; all the authors participated in data analysis and interpretation, and contributed to the writing and editing of the manuscript.

## Conflict of Interest Statement

The authors declare that the research was conducted in the absence of any commercial or financial relationships that could be construed as a potential conflict of interest.

## Footnotes

1 http://rice.plantbiology.msu.edu/

## References

[B1] Abe A. Kosugi S. Yoshida K. Natsume S. Takagi H. Kanzaki H. (2012). Genome sequencing reveals agronomically important loci in rice using MutMap. *Nat. Biotechnol.* 30 174–178. 10.1038/nbt.2095 22267009

[B2] Allen G. C. Flores-Vergara M. A. Krasynanski S. Kumar S. Thompson W. F. (2006). A modified protocol for rapid DNA isolation from plant tissues using cetyltrimethylammonium bromide. *Nat. Protoc.* 1 2320–2325. 10.1038/nprot.2006.384 17406474

[B3] Allen R. S. Nakasugi K. Doran R. L. Millar A. A. Waterhouse P. M. (2013). Facile mutant identification via a single parental backcross method and application of whole genome sequencing based mapping pipelines. *Front. Plant Sci.* 4:362 10.3389/fpls.2013.00362 PMC3772335 24062760

[B4] Austin R. S. Vidaurre D. Stamatiou G. Breit R. Provart N. J. Bonetta D. (2011). Next-generation mapping of Arabidopsis genes. *Plant J.* 67 715–725. 10.1111/j.1365-313X.2011.04619.x 21518053

[B5] Benjamini Y. Speed T. P. (2012). Summarizing and correcting the GC content bias in high-throughput sequencing. *Nucleic Acids Res.* 40:e72 10.1093/nar/gks001 PMC3378858 22323520

[B6] Dohm J. C. Lottaz C. Borodina T. Himmelbauer H. (2008). Substantial biases in ultra-short read data sets from high-throughput DNA sequencing. *Nucleic Acids Res.* 36:e105 10.1093/nar/gkn425 PMC2532726 18660515

[B7] Fatland B. L. Nikolau B. J. Wurtele E. S. (2005). Reverse genetic characterization of cytosolic acetyl-CoA generation by ATP-citrate lyase in *Arabidopsis*. *Plant Cell* 17 182–203. 10.1105/tpc.104.026211 15608338 PMC544498

[B8] Fekih R. Takagi H. Tamiru M. Abe A. Natsume S. Yaegashi H. (2013). MutMap+: genetic mapping and mutant identification without crossing in rice. *PLoS ONE* 8:e68529 10.1371/journal.pone.0068529. PMC3707850 23874658

[B9] Higginson T. Li S. F. Parish R. W. (2003). *AtMYB103* regulates tapetum and trichome development in *Arabidopsis thaliana*. *Plant J.* 35 177–192. 10.1046/j.1365-313X.2003.01791.x 12848824

[B10] Hill J. T. Demarest B. L. Bisgrove B. W. Gorsi B. Su Y. C. Yost H. J. (2013). MMAPPR: mutation mapping analysis pipeline for pooled RNA-seq. *Genome Res.* 23 687–697. 10.1101/gr.146936.112 23299975 PMC3613585

[B11] Jacoby W. G. (2000). Loess: a nonparametric, graphical tool for depicting relationships between variables. *Elect. Stud.* 19 577–613. 10.1016/S0261-3794(99)00028-1

[B12] Jander G. Norris S. R. Rounsley S. D. Bush D. F. Levin I. M. Last R. L. (2002). Arabidopsis map-based cloning in the post-genome era. *Plant Physiol.* 129 440–450. 10.1104/pp.003533 12068090 PMC1540230

[B13] Langmead B. Salzberg S. L. (2012). Fast gapped-read alignment with Bowtie 2. *Nat. Methods* 9 357–359. 10.1038/nmeth.1923 22388286 PMC3322381

[B14] Li H. Durbin R. (2009). Fast and accurate short read alignment with Burrows-Wheeler transform. *Bioinformatics* 25 1754–1760. 10.1093/bioinformatics/btp324 19451168 PMC2705234

[B15] Li H. Handsaker B. Wysoker A. Fennell T. Ruan J. Homer N. (2009). The sequence alignment/map format and SAMtools. *Bioinformatics* 25 2078–2079. 10.1093/bioinformatics/btp352 19505943 PMC2723002

[B16] Li R. Li Y. Fang X. Yang H. Wang J. Kristiansen K. (2009a). SNP detection for massively parallel whole-genome resequencing. *Genome Res.* 19 1124–1132. 10.1101/gr.088013.108 19420381 PMC2694485

[B17] Li R. Yu C. Li Y. Lam T. W. Yiu S. M. Kristiansen K. (2009b). SOAP2: an improved ultrafast tool for short read alignment. *Bioinformatics* 25 1966–1967. 10.1093/bioinformatics/btp336 19497933

[B18] Lochlainn S. O. Amoah S. Graham N. S. Alamer K. Rios J. J. Kurup S. (2011). High Resolution Melt (HRM) analysis is an efficient tool to genotype EMS mutants in complex crop genomes. *Plant Methods* 7:43 10.1186/1746-4811-7-43 PMC3251530 22152063

[B19] Lukowitz W. Gillmor C. S. Scheible W. R. (2000). Positional cloning in Arabidopsis. Why it feels good to have a genome initiative working for you. *Plant Physiol.* 123 795–805. 10.1104/pp.123.3.795 10889228 PMC1539260

[B20] Martin M. (2011). Cutadapt removes adapter sequences from high-throughput sequencing reads. *Embnet J.* 17 10–12. 10.14806/ej.17.1.200

[B21] Nonomura K. Morohoshi A. Nakano M. Eiguchi M. Miyao A. Hirochika H. (2007). A germ cell specific gene of the *ARGONAUTE* family is essential for the progression of premeiotic mitosis and meiosis during sporogenesis in rice. *Plant Cell* 19 2583–2594. 10.1105/tpc.107.053199 17675402 PMC2002623

[B22] Nordstrom K. J. Albani M. C. James G. V. Gutjahr C. Hartwig B. Turck F. (2013). Mutation identification by direct comparison of whole-genome sequencing data from mutant and wild-type individuals using *k*-mers. *Nat. Biotechnol.* 31 325–330. 10.1038/nbt.2515 23475072

[B23] Peters J. L. Cnudde F. Gerats T. (2003). Forward genetics and map-based cloning approaches. *Trends Plant Sci.* 8 484–491. 10.1016/j.tplants.2003.09.002 14557045

[B24] Phan H. A. Iacuone S. Li S. F. Parish R. W. (2011). The MYB80 transcription factor is required for pollen development and the regulation of tapetal programmed cell death in *Arabidopsis thaliana*. *Plant Cell* 23 2209–2224. 10.1105/tpc.110.082651 21673079 PMC3160043

[B25] Phan H. A. Li S. F. Parish R. W. (2012). MYB80, a regulator of tapetal and pollen development, is functionally conserved in crops. *Plant Mol. Biol.* 78 171–183. 10.1007/s11103-011-9855-0. 22086333

[B26] Pu C. X. Ma Y. Wang J. Zhang Y. C. Jiao X. W. Hu Y. H. (2012). Crinkly4 receptor-like kinase is required to maintain the interlocking of the palea and lemma, and fertility in rice, by promoting epidermal cell differentiation. *Plant J.* 70 940–953. 10.1111/j.1365-313X.2012.04925.x 22332708

[B27] Schneeberger K. Ossowski S. Lanz C. Juul T. Petersen A. H. Nielsen K. L. (2009). SHOREmap: simultaneous mapping and mutation identification by deep sequencing. *Nat. Methods* 6 550–551. 10.1038/nmeth0809-550 19644454

[B28] Shen X. Liu H. Yuan B. Li X. Xu C. Wang S. (2011). OsEDR1 negatively regulates rice bacterial resistance via activation of ethylene biosynthesis. *Plant Cell Environ.* 34 179–191. 10.1111/j.1365-3040.2010.02219.x 20807375

[B29] Takagi H. Uemura A. Yaegashi H. Tamiru M. Abe A. Mitsuoka C. (2013). MutMap-Gap: whole-genome resequencing of mutant F2 progeny bulk combined with de novo assembly of gap regions identifies the rice blast resistance gene Pii. *New Phytol.* 200 276–283. 10.1111/nph.12369 23790109

[B30] Uchida N. Sakamoto T. Kurata T. Tasaka M. (2011). Identification of EMS-induced causal mutations in a non-reference *Arabidopsis thaliana* accession by whole genome sequencing. *Plant Cell Physiol.* 52 716–722. 10.1093/pcp/pcr029 21398646

[B31] Wang M. Tang D. Luo Q. Jin Y. Shen Y. Wang K. (2012). BRK1, a Bub1-related kinase, is essential for generating proper tension between homologous kinetochores at metaphase I of rice meiosis. *Plant Cell* 24 4961–4973. 10.1105/tpc.112.105874 23243128 PMC3556969

[B32] Yoshida A. Suzaki T. Tanaka W. Hirano H. Y. (2009). The homeotic gene *long sterile lemma (G1)* specifies sterile lemma identity in the rice spikelet. *Proc. Natl. Acad. Sci. U.S.A.* 106 20103–20108. 10.1073/pnas.0907896106 19901325 PMC2775035

[B33] Zhu Y. Mang H. G. Sun Q. Qian J. Hipps A. Hua J. (2012). Gene discovery using mutagen-induced polymorphisms and deep sequencing: application to plant disease resistance. *Genetics* 192 139–146. 10.1534/genetics.112.141986 22714407 PMC3430530

